# An Evaluation of Universal Grammar and the Phonological Mind^1^

**DOI:** 10.3389/fpsyg.2016.00015

**Published:** 2016-02-08

**Authors:** Daniel L. Everett

**Affiliations:** Department of Arts and Sciences, Bentley UniversityWaltham, MA, USA

**Keywords:** phonology, recursion, universal grammar, linguistic universals, syntax

## Abstract

This paper argues against the hypothesis of a “phonological mind” advanced by Berent. It establishes that there is no evidence that phonology is innate and that, in fact, the simplest hypothesis seems to be that phonology is learned like other human abilities. Moreover, the paper fleshes out the original claim of Philip Lieberman that Universal Grammar predicts that not everyone should be able to learn every language, i.e., the opposite of what UG is normally thought to predict. The paper also underscores the problem that the absence of recursion in Pirahã represents for Universal Grammar proposals.

## Introduction: Two Conceptions of Language

From Panini in India to Plato in Greece, scholars have for centuries studied human language to reveal the essence of human nature (cf. Everett, accepted for recent arguments against the very idea of “human nature”)^2^. Simplifying somewhat, the modern study of language has investigated how languages diverge over time (diachronic linguistics). It examines the physical properties of speech sounds, borrowing from physiology and physics to understand how sounds are made, how they are transmitted across a medium, how they are heard, and what their articulatory and physical properties are both in isolation and in context (phonetics). This scientific tradition has also examined how larger spans of sounds are organized into a phonology (syllables, “feet,” and so on). It also investigates word-formation (morphology), how sentences are put together (syntax), how stories are structured (discourse theory), what meaning is and how it interacts with language forms (semantics), and how language is shaped via the apex of human linguistic development – conversations (pragmatics). And it asks about the universality of its findings.

From this rich history of linguistic studies, we have reached a divide: some researchers believe that language structures emerge from universal principles of grammar. For the former, as early as the late 17th century, culminating in the Port Royal grammar of 1660, linguists and philologists began to postulate a universal base for human languages. Such researchers made the case that all languages likely trace back to some original blueprint. Then in the latter half of the 20th century, Noam Chomsky took on the challenge of understanding and investigating this blueprint, by looking to biology as the source of grammar, proposing that all languages are simply local manifestations of a biologically transmitted Universal Grammar.

Other researchers believe that grammar is in essence a local phenomenon. This is the alternative I explore in what follows. In this view language and its components (grammar, phonetics, phonology, semantics, and so on) are perceived as local, cultural-communicational outputs, with little or no evidence for a genetic blueprint for grammar. I largely approach this issue negatively in what follows, arguing against UG proposals, from the perspective of Everett (2012a; see also Everett, accepted, in progress, among others). I argue that there is no evidence for UG, not even from the most articulated grammatical proposals in its favor to date, Hauser et al. (2002) and Berent (2013a), along the way indirectly supporting my own theory (see references) that language is a tool shaped by culture (among other things) for communication. This is by no means a novel position, though it is a path less followed. It represents in fact the traditional position of the most influential North American linguists of the early 20th century, Franz Boas and Edward Sapir. Much of what follows draws heavily from arguments and texts provided in Everett (accepted), updated where appropriate.^3^

## “Man-in-a-Can” View of Language

The modern idea of Universal Grammar (UG) emerged from Chomsky’s work. The basic thesis of UG is that there is something about the genetic component of human nature that guarantees that there will be a core of “knowledge” common to all humans. If so, then languages are essentially the same and only superficially different.^4^

Yet an often overlooked, genetic criticism of UG, raised by Lieberman (2013, 56ff) is that UG predicts the opposite of what it is claimed to predict. UG was proposed to account for a hypothesized (never demonstrated) acquisitional homogeneity, which children across cultures are said to achieve for their native languages, as well as for the fact that all languages are built on the same grammatical plan, a plan located somehow, somewhere in the human genome (in a way that has never been specified in the literature). As Lieberman points out, however, if language were actually specified on the genes, it would be subject to mutations, presenting a non-trivial problem for UG.

To take a concrete example, consider one commonly assumed “parameter” of UG, the so-called “pro-drop” parameter – intended to account for the ability of speakers of a language to omit overt subjects from sentences. Thus in Portuguese, a pro-drop language via a single gene (unlikely) or relationships among multiple genes, one can utter “Está chovendo” while in English the literal translation *Is raining* is ungrammatical. Instead, English speakers must say “It is raining,” for the reason that English apparently requires subjects and thus lacks “pro-drop.”^5^ The question that arises is whether it is possible for there to be a mutation that would prevent a particular person from learning a pro-drop language. Such a mutation might subsequently spread through a population via genetic drift or some such, though that is not crucial. We need only to find a single individual that cannot learn English-like or Portuguese-like languages, with no other cognitive deficit – (simplifying) if we assume that pro-drop is a genetically based parameter. This is a valid question to put to any nativist theory. In fact, rather than view this negatively, it can be seen positively – a strong prediction by the theory of UG.^6^ It would strongly support UG to find an individual or a population whose only “cognitive quirk” were the inability to learn pro-drop.^7^

A UG-proponent might rebut this argument, however, by claiming that the “language instinct” is an organ and is no more subject to mutation than arms, legs, livers, hearts, etc. But all *are* subject to mutations. There are many genetic disorders of the body and brain (e.g., sickle-cell anemia, dwarfism, autism, and so on). Such disorders are usually fatal or produce reductions in offspring and thus are not selected for. Sickle-cell anemia, for example, shortens the lives of carriers relative to healthy people (not good) but lengthens it relative to people stricken with malaria (good). It spreads through a population in spite of the unpleasant end it brings to its hosts, because it nonetheless provides local advantages. In language a local advantage might be to learn one’s parents’ language more quickly, even at the expense of being able to learn other languages. There are indeed mutations responsible for people being born with different genes for body shape, etc. In other words, and quite ironically, if grammar is carried on the genes, then the strongest evidence for UG would be the discovery that *not all people may be able to learn every language*.^8^

There are claims for some mutations in language by proponents of UG, but these are not the same. For example, consider the following quote from Reboul (2012, p. 312):

“I would like to end this paper by discussing one of Everett’s claims regarding the non-biological nature of language... if language is biological, one would expect to find “culture-gene mutations affecting specific languages of the world” (Everett, 2012a, p. 42) and these do not exist. In fact, recent findings (see Dediu and Ladd, 2007; Nettle, 2007) suggest that such mutations exist. Dediu and Ladd established a strong correlation between (geographically dispersed) tone-languages and allele frequencies for two genes (ASPM and Microcephalin) in the populations speaking those languages as compared with speakers of non-tonal languages. The interpretation is that these specific alleles would facilitate the learning of tonal languages through better acoustic discrimination.”

The idea that language (I-language, grammar, etc.) is carried by the genes definitely predicts that it is subject to mutations. This is not an argument – it is an *entailment* of nativist theory. And cultures, as Everett (2012a) points out, provide one source of selectional pressure. What counts against nativism of the Chomskyan variety is the clear failure of this prediction. Moreover, the “counterexample” to my claim that Reboul provides merely strengthens my case.

The claim that Reboul is supposed to be criticizing is the idea that one population could, through selection of some genetic features of language, be unable to learn the language of another population. The findings of Dediu and Ladd, if true (and I doubt it), far from falsifying my claim support it. This is because they show that evolution can enhance perception by human populations of the phonological/phonetic forms that they commonly use. Their results apparently show that some populations speaking tonal languages become better at perceiving tones than others. But this contradicts my claim not at all, because *all* languages use pitch. Therefore, this enhancement would benefit all speakers of all populations and could not become the basis for one population losing the ability to learn the language of another. However, this gets us back to our original question. If this tonal restriction were indeed an example of a cultural (speaking a tone language) pressure affecting one’s genes, then the absence of the opposite effect, the principle prediction of Chomskyan Universal Grammar – a genetic mutation that would render one population unable to learn the grammar of another – becomes even more mysterious.

Unfortunately, the most serious problem for UG is that as the years have passed, it has reached the point that it is vague and it makes no predictions about language proper – it is disconnected from empirical content. For example in response to a now famous paper by Evans and Levinson (2009), “The Myth of Language Universals,” the UG community objected to the idea that UG predicts universals in Evans and Levinson’s “naive” sense. Critics claimed that Evans and Levinson confused UG with Greenbergian universals (as discussed below).

To give a closer-to-home, concrete illustration of a lack of empirical constraints on the content of Chomskyan linguistics, let’s look at the so-called Pirahã recursion debate. I have in past publications (see especially Everett, 2005, Everett, 2012a,b) criticized Noam Chomsky’s claim that all languages are built on a recursive grammatical procedure he calls “Merge,” defined as in (1):

(1) Merge (α, β) → {α, {α, β}}.

If α is a verb, e.g., ‘eat’ and β a noun, e.g., ‘eggs,’ then this will produce a verb phrase (i.e., where alpha is the head of the phrase), ‘eat eggs.’ As I said in Everett (2012b), “The operation Merge incorporates two highly theory-internal assumptions that have been seriously challenged in recent literature (see Everett, accepted, in progress). The first is that all grammatical structures are binary branching, since Merge can only produce such outputs. The second is that Merge requires that all syntactic structures be endocentric (i.e., headed by a unit of the same category as the containing structure, e.g., a noun heading a noun phrase a verb a verb phrase, etc.).

My criticism is based on the fact that the Amazonian language, Pirahã, among others (see Kornai, 2014; Jackendoff and Wittenberg, in preparation), lacks recursive structures (Everett, 2005, Everett, 2012b; Futrell et al., in preparation) – and thus, a fortiori, Merge. My claim is that the absence of recursion is the result of cultural values, rather than a culture-independent grammar. One of the most common objections raised to this criticism of Chomskyan theory is that the superficial appearance of lacking recursion in a language does not necessarily mean that the language could not be derived from a recursive process like Merge. There are ways to rescue the theory. And of course this is correct.

From this latter observation, some conclude that the (misguided in their perspective) suggestion that Piraha represents a problem for Chomskyan theory is due to the failure distinguish between Greenbergian vs. Chomskyan universals. Greenbergian universals (Greenberg, 1966) have always referred to linguistic phenomena that can actually be observed (and thus easily falsified). These claims are tightly constrained empirically.

On the other hand, Chomskyan universals are quite different because they are never directly observable. Chomsky’s concept of universals is that they are restrictions on language development, not necessarily observed directly in actual surface structures of languages. Formal universals are grammatical principles or processes or constraints common to all languages – that is, supposedly following from UG – at some level of abstraction from the observable data. These abstractions can only be appreciated, it seems, by the appropriate theoretician. Unfortunately, this makes formal universals difficult to falsify because they can always be saved by abstract, unseen principles or entities, e.g., so-called “empty categories.”

In this sense, the Chomskyan claim regarding recursion (Hauser et al., 2002) would be that all languages are formed by a recursive process, even though the superficial manifestation of that process may not look recursive to the untrained eye. A language without Merge would lack utterances of more than two words according to Chomsky (by this strange reasoning, all utterances greater than three words would support Chomsky, a rather low threshold of evidence). So long as we can say that a sentence is the output of Merge, limited in some way, then it was produced recursively, even though superficially non-recursive. The Greenbergian perspective, on the other hand, would be that either you see recursion or it is not there.

Both positions are completely rational and sensible. But, as I have said, the Chomskyan view renders the specific claim that all languages are formed by Merge untestable. In Chomsky’s earlier writings he claimed that if two grammars produce the same surface strings (weak generative capacity), we could still test them by examining the predictions of the structures they predict for the strings (strong generative capacity). Since my work on Piraha recursion (as well as Wari’; Everett and Kern, 1997; Everett, 2009) has shown that the predictions Merge makes are problematic (falsified if that were possible with such abstract universals), I have dealt exclusively with strong generative capacity. On the other hand, a linguist could add ancillary hypotheses to their accounts in order to save Merge, entailing two consequences: (i) Merge loses all predictive power and (ii) Merge provides a longer, hence less parsimonious, account of the same structures (Everett, 2012b).

Nativism, again, is the idea not only that we are innately capable of language (everyone surely believes this), but that our capabilities are specific to particular domains, e.g., grammar. Now veterinarians who artificially inseminate animals, such as thoroughbreds or other competitive breeds, occasionally refer to their metal-encased syringes of semen as “man-in-a-can.” This is a good metaphor for some theories like UG, which place the development of human abilities in the genes rather than the environment, i.e., those that lean strongly to the nature side of the nature-nurture continuum, predicting that all languages emerge from the same biological can.

Though I have argued (Everett, 2012a) that there is no convincing evidence for UG from universals, acquisition, nor language deficits, some have countered such arguments by claiming that “emerging” languages (creoles, Nicaraguan Sign Language, Homesigns, and so on) manifest UG principles that could not have been learned. Everett (2012a, 2015) argues that they show nothing of the sort.

Stepping back a bit, it is clear that all creatures have instincts or innate capacities. Even so, the evidence presented for such capacities is often weak. This is particularly true for claims on cognitive nativism. In fact, if Everett (accepted) is correct, then higher-level cognitive capacities in *Homo sapiens* are the least likely places to find instincts. If one is claiming that a cognitive characteristic is innate or an instinct, they must do the following at a minimum:

(1) Show evidence for something that doesn’t seem to be learnable.
(2) Argue convincingly that it cannot be learned from the womb to the time of testing.
(3) Define “innate” or “instinct” so as to encompass not merely “bias” or “capacity” but also “knowledge.”
(4) Provide a plausible account of the evolution of the trait.
(5) Keep genetics and epigenetics (constraints – embryological, environmental – on the strength, absence, or presence of genetic effects) separate.
(6) Devise a methodology more sound than babies’ sucking or eye-movements for investigating cognitive characteristics (Clearfield and Mix, 2001).

If you can’t meet these minimal requirements, talk of instincts, UG, nativism, etc. is premature. Yet because almost no claim for instincts gets beyond 1, as Blumberg (2006, p. 205) says, such talk is “bedtime stories” for adults (see also: http://www.pointofinquiry.org/mark_blumberg_freaks_of_nature/).

What does this mean? It means that if you see claims for a morality instinct, an art instinct, a language instinct, etc. you are reading nothing more speculation, unless it gets significantly beyond level 1 above. I am not aware of any that do.

To offer a more detailed example of the shortcomings of UG proposals, let’s consider the research program developed by Iris Berent on “the phonological mind.” My theory of “dark matter” (Everett, accepted) implies that instincts should be minimized in *Homo sapiens*. This is not because instincts are incompatible with culture or dark matter as defined in Everett (accepted). Rather, they simply become less relevant to our understanding. If humans learn from and participate in their surroundings and language, then it turns out that instincts become less compelling (Prinz, 2012, 2013). Of course, the concept of instincts is common enough in the literature on animal behavior, in Evolutionary Psychology, as well as in Chomskyan linguistics. At the same time, everyone agrees general learning is responsible for at least some of how people come to learn about the world, their society, and themselves. My claim (Everett, accepted) is that, given our capacity for general learning, that instincts complicate the picture of human development, going against the inherent cognitive and cerebral plasticity of the species. In my view, appeal to epistemological nativism should be excised by Occam’s Razor.

In what follows, I want to give a concrete example of what I mean by discussing and rejecting recent work on phonological nativism (Berent, 2013a). To anticipate somewhat, the problems faced by all nativist proposals include the following: (i) the non-linear relationship of genotype to phenotype; (ii) failure to link “instincts” to environment – today’s instincts are often tomorrow’s learning, once we learn more about the environmental pressures to acquire certain knowledge; (iii) problematic definitions of innateness; (iv) failure to rule out learning before proposing an instinct; (v) the unclear content of what is left over for instincts after acquired dark matter (all tacit knowledge) is accounted for; (vi) lack of an evolutionary account for the origin of the instincts.

In Berent’s (2013a) *The Phonological Mind*, the author argues in detail for apparently innate preferences for some sounds and sound sequences (and signs and sign sequences) in all languages. I want to briefly review the more detailed criticisms of Everett (accepted) of her proposals, limited to a small portion of her monograph.^9^ From the outset we should observe that the most serious shortcomings of her notion of innate phonological knowledge, in fact a problem for all nativist theories, is the “origin problem.” The question needing to be answered is “Where did the phonological knowledge come from?” Without an account of the *evolution of an instinct*, proposing nativist hypotheses is pure speculation. Rather, at best, we can take non-evolutionary evidence for an instinct as explanada rather than explanans. Berent’s specific proposal is that her experimental results from English, Spanish, French, and Korean support her proposal that there is knowledge of some type that leads to a sonority sequencing generalization (SSG) inborn in all *Homo sapiens*.

**FIGURE 1:**
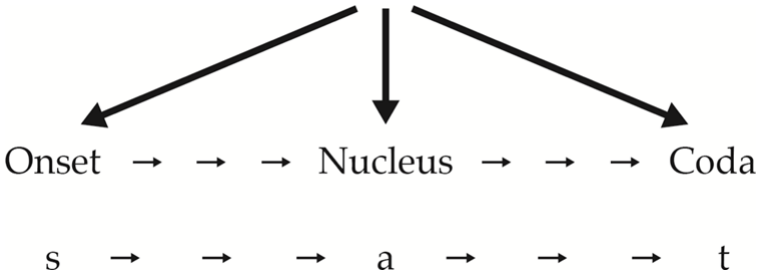
**Syllable structure one**.

To understand her arguments, however, we must first understand the terms she uses, beginning with “sonority.” Sonority is the property of one sound being inherently louder than another sound. For example, when the vowel [a] is produced in any language the mouth is open wider than for other vowels and, like other vowels, [a] offers very little impedance to the flow of air out of our lungs and mouths. This makes [a] the loudest sound relatively speaking of all phonemes of English. A sound with less inherent loudness, e.g., [k] is said to be less sonorous. Several of Berent’s experiments demonstrate that speakers of all the languages she tested, children and adults, prefer words organized according to the SSG. The idea behind the SSG is that the least loud (sonorous) segments are found at the far edges of syllables while the loudest segments are found closer to the nucleus of the syllable. To see what is meant more clearly, consider a single syllable work (monosyllable) such as “sat,” whose structure would be as shown in **Figure 1**.

Since [a] is the most sonorous element, it is in the nucleus position. [s] and [t] are at the margins, onset and coda, as they should be. Now take the hypothetical syllables, [bli] and [lbi].

**FIGURE 2:**
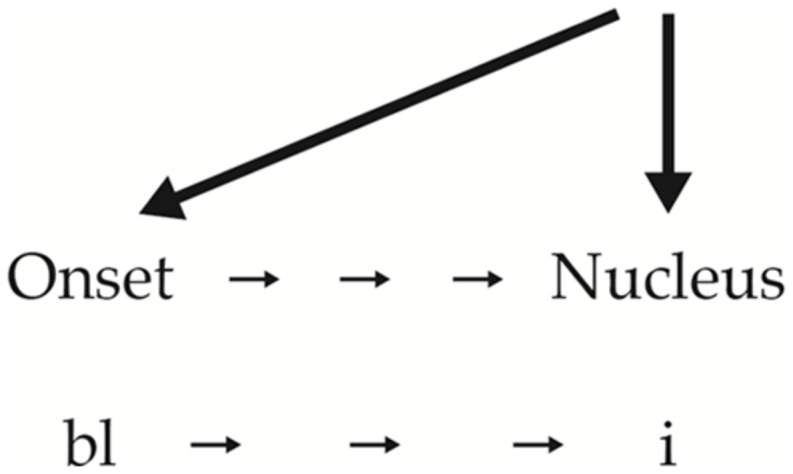
**Syllable structure two**.

Both [bli] and [lbi] have what phonologists refer to as “complex onsets,” multiple phonemes in a single onset the same can happen with codas as with “pant” in which [n] and [t] form a complex coda. Now, according to the SSG, since [b] is less sonorous than [l], it should come first in the onset. This means that [bli] is as a well-formed syllable should be, i.e., organized from least sonorant/sonorous segment to most sonorous, [i], and then, if there were a coda, to a segment less sonorous than [i] (softer → louder). Therefore, the correct syllabic organization is shown in the following diagram (**Figure 2**).

Such preferences emerge even when the speakers’ native languages otherwise allow grammatical strings which appear to violate the SSG. Since the SSG is so important to the work on a phonological instinct, we need to take a closer look at it. To make it concrete, let’s consider one proposal regarding the so-called sonority hierarchy (as we will see, not only do many phoneticians consider this hierarchy to be a spurious observation, but it is also inadequate to account for many phonotactic generalizations, suggesting that not sonority but some other principle is behind Berent’s experimental results).^10^ One form of this hierarchy comes from Selkirk (1984; from most sonorant on left to least on right):

[a] > [e o] > [i u] > [r] > [l] > [m n ŋ] > [z v ð] > [s f θ] > [b d g] > [p t k].

The hierarchy has often been proposed as the basis for the SSG, which might also be thought of as organizing syllables left to right into a crescendo, peak, and decrescendo of sonority, going from the least sonorant (least inherently loud) to the most sonorant (most inherently loud) and back down, in inverse order, to the least sonorant (in fact, I was once a proponent of the SSG myself. See Everett (1995, for a sustained attempt to demonstrate the efficacy of this hierarchy in organizing Banawá syllable structure).

Without reviewing all of her experimental results (which all roughly show the same thing – preference in subjects for the SSG in some conditions), consider the following evidence that Berent (2013b, p. 322) brings to bear:

“... Syllables with ill-formed onsets (e.g., lba) tend to be systematically misidentified (e.g., as leba)—the worse formed the syllable, the more likely the misidentification. Thus, misidentification is most likely in lba followed by bda, and is least likely in bna. Crucially, the sensitivity to syllable structure occurs even when such onsets are unattested in participants’ languages, and it is evident in adults [64,67–70,73] and young children...”

Again, as we have seen, a licit syllable should build from least sonorant to most sonorant and then back down to least sonorant, across its onset, nucleus, and coda. This means that while [a] is the ideal syllable nucleus for English, a voiceless stop like [p, t, k] would be the least desirable (though in many languages this hierarchy is violated regularly, e.g., Berber). Thus a syllable like [pap] would respect the hierarchy, but there should be no syllable like [opa] (though of course there is a perfectly fine *bisyllabic* German word *opa* “grandpa”). For the latter word, the SSG would only permit this to be syllabified as two syllables [o] and [pa] with each vowel its own syllable nucleus. This is because both [o] and [i] are more sonorous than [p] so [p] must be either the coda or the onset of a syllable in which one of these two vowels is the nucleus.^11^ Moreover, according to the SSG, a syllable like [psap] should be favored over a syllable [spap]. This gets us to the obvious question of why “misidentification” by Korean speakers is least likely in *bna* (even though Korean itself lacks such sequences)? Because, according to Berent, all humans are born with an SSG instinct.

I do not think anything of the kind follows. To show this, I first want to argue that there is no SSG period, not phonetically, grammatically, or even functionally. Second, I argue that even if we ignored the first argument, i.e., even if some other, better (though yet undiscovered) principle than the SSG were appealed to, the arguments for a phonology instinct do not go through. Third, I offer detailed objections to every conclusion she draws from her work, concluding that there is no such thing as the “phonological mind.”

Let’s address first the reasons behind the claim that the SSG is not an explanation for phonotactics. The reasons are three: (i) there is no phonetic or functional basis for the generalization; (ii) the SSG that Berent appeals to is too weak – it fails to capture important, near-universal phonotactic generalizations; (iii) the generalization is too strong – it rules out commonly observed patterns in natural languages, e.g., English, that violate it. But then if the SSG has no empirical basis in phonetics or phonology and is simply a spurious observation, it is unavailable for grammaticalization and therefore cannot serve as the basis for the evolution of an instinct (though, of course, some other concept or principle might be). One might reply that if the SSG is unable to explain all phonotactic constraints, that doesn’t mean that we should throw it out. Perhaps we can simply supplement the SSG with other principles. But why accept a disjointed set of “principles” to account for something that may have an easier account based more solidly in phonetics and perception? Before we can see this, though, let’s look at the SSG in more detail.

The ideas of sonority and sonority sequencing have been around for centuries. Ohala (1992) claims that the first reference to a sonority hierarchy was in 1765. Certainly there are references to this in the nineteenth and early twentieth centuries. As Ohala observes, however, references to the SSG as an explanation for syllable structure are circular, descriptively inadequate, and not well-integrated with other phonetic and phonological phenomena.

According to Ohala, both the SSG and the syllable itself are theoretical constructs that lack universal acceptance. There is certainly no complete phonetic understanding of either, a fact that facilitates circularity in discussing them. If we take a sequence such as *alba*, most phonologists would argue that the word has two syllables, and that the syllable boundary must fall between /l/ and /b/, because the syllable break a.lba would produce the syllable [a], which is fine, but also the syllable [lba] which violates the SSG ([l] is more sonorous than [b] and thus should be closer to the nucleus than [b]). On the other hand, if the syllable boundary is al.ba, then both syllables respect the SSG, [al] because [a] is a valid nucleus and [l] a valid coda and [ba] because [b] is a valid onset and [a] is a valid nucleus. The fact that [l] and [b] are in separate syllables by this analysis means that there is no SSG violation, which there was in [a.lba]. Therefore, SSG guides the parsing (analysis) of syllables. However, this is severely circular if the sequences parsed by the SSG then are used again as evidence for the SSG.

The SSG is also descriptively inadequate because it is at once too weak and too strong. For example, most languages strongly disprefer sequences such as /ji/, /wu/, and so on, or, as Ohala (1992, p. 321) puts it “... offglides with lowered F2 and F3 are disfavored after consonants with lowered F2 and F3.”^11^^,^^12^ Ohala’s generalization here is vital for phonotactics crosslinguistically and yet it falls outside the SSG, since the SSG allows all such sequences. This means that if a single generalization or principle, of the type Ohala explores in his article, can be found that accounts for the SSG’s empirical range plus these other data, it is to be preferred. Moreover, the SSG would then hardly be the basis for an instinct and Berent’s experiments would be merely skirting the edges of the real generalization. As we see, this is indeed what seems to be happening in her work. The SSG simply has no way of allowing a *dw* sequence, as in *dwarf* or *tw* in *twin* while prohibiting *bw*. Yet [dw] and [tw] are much more common than [bw], according to Ohala (though this sequence is observed in some loanwords, e.g., *bwana*), facts entirely missed by the SSG.

Unfortunately, Berent neither notices the problem that such sequences raise for the SSG “instinct” nor does she experimentally test the SSG based on a firm understanding of the relevant phonetics. Rather, she assumes that since the SSG is “grammaticalized” and now an instinct the phonetics no longer matter. But this is entirely circular. Here, the lack of phonetic experience and background in phonological analysis seem to have led to hasty acceptance of the SSG, based on the work of a few phonologists, without careful investigation of its empirical adequacy. This is a crucial shortcoming when it comes to imputing these behaviors to “core knowledge” (knowledge that all humans are hypothesized to be born with). It hardly needs mentioning, however, that a spurious observation of a few phonlogists is not likely to serve as an instinct.

To take another obvious problem for the SSG, sequences involving syllable-initial sibilants are common crosslinguistically, even though they violate the SSG. Thus the SSG encounters problems in accounting for English words like “spark,” “start,” “skank,” etc. Since [t], [k], [p] – the voiceless stops – are not as loud/sonorous as [s], they should come first in the complex onset of the syllable. According to the SSG, that is, [psark], [tsart], should be grammatical words of English (false) while [spark], [start], etc. should be ungrammatical – also false. Thus the SSG is too strong (incorrectly prohibits [spark]) and too weak (incorrectly predicts [psark]) to offer an account of English phonotactics. Joining these observations to our earlier ones, we see that the SSG not only allows illicit sequences such as /ji/ while prohibiting perfectly fine sequences such as /sp/, it simply is not up to the task of English phonotactics more generally. And although many phonologists have noted such exceptions, there is not way to handle them except via ancillary hypotheses (think “epicycles”) if the basis of one’s theory of phonotactics is the SSG.

I conclude that Berent’s phonology instinct cannot be based on the SSG, because the latter doesn’t exist. She might claim instead that the instinct she is after is based on a related principle or that the SSG was never intended to account for all of phonotactics, only a smaller subset, and that phonotactics more broadly require a set of principles. Or we might suggest that the principles behind phonotactics are not phonological at all, but phonetic, having to do with relative formant relationships, along the lines adumbrated by Ohala. But while such alternatives might better fit the facts she is invested in, a new principle or set of principles cannot rescue her proposal. This is because the evidence she provides for an instinct fails no matter what principle she might appeal to. To see why let’s consider what Berent infelicitously refers to (Berent, 2013b, p. 320) as “the seven wonders of phonology.” She takes all of these as evidence for “phonological core knowledge.” I see them all as red herrings, rather than as evidence for a phonological mind or an instinct. These “wonders” are:

(1) Phonology employs algebraic rules;
(2) Phonology shows universal constraints or rules, e.g., the SSG;
(3) Phonology shows shared design of all phonological systems;
(4) Phonology provides useful scaffolding for other human abilities;
(5) Phonological constraints such as the SSG show early ontogenetic onset;
(6) Phonology shows a unique design unlike other cognitive domains;
(7) Phonology shows regenesis – phonological systems, e.g., sign languages, created *de novo* always draw on the same principles – they never emerge ex nihilo.

These are worth exploring, however, because Berent’s work is a model for other claims of grammatical innateness and far better articulated than most. Therefore, let’s consider each of them in turn.

“Algebraic rules” are nothing more than the standard rules that linguists have used since Panini (4th century BCE). For example, Berent uses an example of such a rule that she refers to as the “AAB rule” in Semitic phonologies. In Semitic languages, as is well-known, consonants and vowels mark the morphosyntactic functions of words, using different spacings and sequences (internal to the word) of Cs or vs. based on conjugation or *binyanim* – the order of consonants and intercalated vowels. An example of what the variables here are illustrated below:

Modern Hebrew

CaCaC katav ‘write’
niCCaC niršam ‘register’
hiCCiC himšix ‘continue’
CiCeC limed ‘teach’
hitCaCeC hitlabeš ‘get dressed.’

In other languages such functions would most frequently be marked by suffixes, infixes, prefixes, and so on. So, clearly, taking only this single, common example, variables are indeed found in phonological rules.

Now, in Berent’s AAB rule (more precisely, it should be stated as a constraint “^∗^AAB,” where ^∗^ indicates that the sequence AAB is ungrammatical) is designed to capture the generalization that the initial consonants of a word cannot be the same. Thus a word like ^∗^*sisum* would be ungrammatical, because the first two consonants are /s/ and /s/, violating the constraint. The constraint is algebraic because A and B are variables ranging across different phonological features (though A must be a consonant). But calling this an algebraic rule and using this as evidence for an instinct makes little sense. Such rules are regularly learned and operate in almost every are of human cognition. For example, one could adopt a constraint on dining seating arrangements of the type ^∗^G_1_G_1_X, i.e., the first two chairs at a dinner table cannot be occupied by people of the same gender (G), even though between the chairs there could be flower vases, etc. Humans learn to generalize across instances, using variables frequently. Absolutely nothing follows from this regarding instincts.

Universality is appealed to by Berent as further evidence for a phonology instinct. But as any linguist can affirm (especially in light of controversies over how to determine whether something is universal or not in modern linguistic theory), there are many definitions, uses, and abuses of the term “universality” in linguistics. For example, some linguists, e.g., Greenberg (1966) and Evans and Levinson (2009) argue that for something to be meaningfully universal, it actually has to be observable in every language. That is, a universal is a concrete entity. If it is not found in all languages, it is not universal. That is simple enough, but some linguists, e.g., Chomsky (1986), prefer a more abstract conception of universal such that for something to be universal it need only be available to human linguistic cognition. This set of universal affordances is referred to as the “toolbox.” I have argued against this approach in many places, for being imprecise and often circular (in particular Everett, 2012a,b). But in any case, Berent clearly follows the notion of “universal” advocated by Chomsky and Jackendoff, inter alia. Such universals need not be observed in all languages. Thus Berent would claim that the SSG is universal, not because it is obeyed in all its particulars in every language – like me, she would recognize that English allows violations of the SSG – but because her experiments with speakers of various languages show that they have preferences and so on that seem to be guided by knowledge of the SSG, even when their own native languages do not follow the SSG in particulars or have a simple syllable structure that is by definition unable to guide their behavior in experiments. If a Korean speaker, for example, shows preference for or perceptual illusions with some onset clusters and not others – in spite of the fact that there are no such clusters in Korean (and thus s/he could not have learned them, presumably), then this shows the universality of the SSG (as part of the linguistic toolbox).

But there is a huge leap taken in reasoning from this type of behavior to the presence of innate constraints on syllable structure. For example, there are phonetic reasons why Korean (or any) speakers prefer or more easily perceive, let us say, [bna] sequences rather than [lba], even though neither sequence is found in Korean. One simple explanation that comes to mind (and highlighted by phoneticians, though overlooked by many phonologists), is that the sequence [bna] is easier to perceive than [lba] because the interconsonantal transition in the onset of the former syllable produces better acoustic cues than in the second. Berent tries to rule out this kind of interpretation by arguing that the same restrictions show up in reading. But reading performance is irrelevant here for a couple of reasons. First, we know too little about the relationship between speaking and reading cognitively to draw any firm conclusions about similarity or dissimilarities in their performance to use as a comparison, in spite of a growing body of research on this topic. Second, in looking at new words speakers often try to create the phonology in their heads and so this “silent pronunciation” could guide such speakers’ choices, etc. Everyone (modulo pathology) has roughly the same ears matched to roughly the same vocal apparatus. Thus although phonologies can grammaticalize violations of functionally preferable phonotactic constraints, one would expect that in experiments that clearly dissociate the experimental data from the speaker’s own language, the functionality of the structures, e.g., being auditorily easier to distinguish, will emerge as decisive factors, accounting for speakers’ reactions to non-native sequences that respect or violate sonority sequencing, etc. In fact, there is a name for this, though with a somewhat different emphasis, in Optimality Theoretic Phonology (Prince and Smolensky, 1993/2004; McCarthy and Prince, 1994) – the “emergence of the unmarked.” So there is nothing special I can see about the universality of these preferences. First, as we have seen, the SSG is not the principle implicated here, because there is no such principle. It is a spurious generalization. Second, local phonologies may build on cultural preferences to produce violations of preferable phonetic sequences, but the hearers are not slaves to these preferences. Let us say that a language has a word like “lbap.” In spite of this, the phonetic prediction would be that in an experimental situation, the speakers would likely prefer “blap” and reject “lbap,” since the former is easier to distinguish clearly in a semantically or pragmatically or culturally neutral environment. In other words, when asked to make judgments in an experiment about abstract sequences, it is unsurprising that the superiority of the functionality of some structures emerges as decisive. Such motivations reflect the fact that the ear and the vocal apparatus evolved together. Therefore, what Berent takes to be a grammatical and cognitive universal is neither, but rather a fact about perceptual ability, unrelated to a phonology instinct.

Next, Berent talks about “shared design.” This is just the idea that all known phonological systems derive from similar phonological features. But this is not a “wonder” of any sort. There is nothing inherently instinctual in building new phonological systems from the same vocal apparatus and auditory system, using in particular the more phonetically grounded components of segmental sequencing.

Another purported “wonder” is what Berent refers to as “scaffolding.” This is nothing more than the idea that our phonologies are reused. They serve double duty – in grammar and as a basis for our reading and writing (and other related skills). This is of course false in much of some writing systems (e.g., Epi-Olmec hieroglyphics, where speaking and writing are based on nearly non-overlapping principles). In fact “reuse” is expected in cognitive or biological systems to avoid unnecessary duplication of effort. It is not only a crucial feature of brain functioning (Anderson, 2014), but it is common among humans to reuse technology – e.g., the use of cutting instruments for a variety of purposes, from opening cans to carving ivory. Therefore, reuse is a common strategy of cognition, evolution, resource management, and on and on, and is thus orthogonal to the question of instincts.

Next, Berent talks about “regenesis,” the appearance of the same (apparently) phonological principles in new languages, in particular when principles of spoken phonology, e.g., the SSG according to Berent, show up in signed systems. The claim is that the SSG emerges when humans generate a new phonological system *de novo*. But even here, assuming we can replace the invalid SSG with a valid principle, we must use caution in imputing “principles” to others as innate knowledge. We have just seen, after all, how the particular phonetic preference Berent calls the SSG could occur without instincts.

But even if we take her claims and results and face value, “regenesis” still offers no support for nativism. In spoken languages, the notion simply obscures the larger generalization or set of generalizations that *people always prefer on the best-sounding sequences perceptually*, even when cultural effects in their native languages override these. Berent again attempts to counter this with research on sequences of signs in signed languages. Yet there is no sound-based principle in common between signed and spoken languages – by definition, since one lacks sounds altogether and the other lacks signs. Both will of course find it useful to organize word-internal signs or sounds to maximize their perceptability, but no one has ever successfully demonstrated that signed languages have “phonology” in the same sense as spoken languages. In fact, I have long maintained that, in spite of broadly similar organizational principles, sign organization in visual vs. spoken languages are grounded in entirely different sets of features (for example, where is the correlate of the feature “high tone” or F2 transition in signed languages?) and thus that talking of them both as having “phonologies” is nothing more than misleading metaphor.

Another “wonder” Berent appeals to show that phonology is an instinct is the common poverty of the stimulus argument or what she refers to as “early onset.” Children show the operation of sophisticated linguistic behaviors early on, so early in fact that a particular researcher might not be able to imagine how it might have been learned, jumping to the conclusion that it must not have been learned but emerges from the child’s innate endowment. Yet all Berent shows in discussing early onset is the completely unremarkable fact that children rapidly learn and prefer those sound sequences that their auditory and articulatory apparatuses have together evolved to recognize and produce most easily. This commonality is not linguistic *per se*. It is physical, like not trying to pick up a ton of bricks with only the strength in one’s arms. Or, more appropriately, in not using sounds that people cannot hear, e.g., with frequency that only dogs can hear.

Finally, Berent argues for “core phonological knowledge” based on what she terms “unique design.” This means that phonology has its own unique properties. But this shows nothing about innate endowment. Burrito-making has its own unique features, as does mathematics, both eminently learnable (like phonology). Berent’s discussion fails to explain whey these unique features could not have been learned, nor why the would be any evolutionary advantage such that natural selection would favor them.

Summing up to this point, Berent has neither established that speakers are following sonority organization that is embedded in their “core knowledge,” nor that her account is superior to more intuitively plausible phonetic principles. Nor are any of her “seven wonders of phonology” remotely wondrous.

And yet, in spite of all of my objections up to this point, there is a far more serious obstacle to accepting the idea of a phonological mind, mentioned at the outset of this discussion. This is what Blumberg (2006) refers to as the problem of “origins” which we have mentioned and which is discussed at length in several recent books (Blumberg, 2006; Buller, 2006; Richardson, 2007; among others) – an obstacle Berent ignores entirely – an all too common omission from proponents of behavioral nativism. Put another way, how could this core knowledge have evolved? More seriously, relative to the SSG, how could an instinct based on any related principle have evolved? As we have seen, to answer the origins problem, Berent would need to explain (as Tinbergen, 1963 among others, discusses at length) the survival pressures, population pressures, environment and so on at the time of the evolution of a valid phonotactic constraint – if the trait appears as a mutation in one mind what leads to its genetic spread to others in a population – what was its fitness advantage? In fact, the question doesn’t even make sense regarding the SSG, since there is no such principle. But even if a better-justified generalization could be found, coming up with any plausible story of the origin of the principle is a huge challenge, as are definitions of innate, instinct, and the entire line of reasoning based on innate knowledge, inborn dark matter.

## Conclusion

In this paper I have argued for three points: first, UG makes only one ironic prediction: *not all people should be able to learn all languages*. Second, the most recent incarnation of UG – recursion (Hauser et al., 2002) – is either falsified or it has no empirical content.^14^ Third, I argue that arguably the most well-developed case for grammatical nativism, Berent (2013a), itself fails to offer convincing evidence for grammatical nativism. Because of the importance and novelty of Berent’s arguments, I have spent the majority of the space allotted arguing against her concept of a “phonological mind.”

## Conflict of Interest Statement

The author declares that the research was conducted in the absence of any commercial or financial relationships that could be construed as a potential conflict of interest.

## Footnotes

2 Of course, the idea that there is no human nature is not original with me. See, e.g., Marx’s (1845/1976) “Thesen über Feuerbach."

3 There are many other sources, however. Evans (2014) is another important source, one of the very best, of arguments against Universal Grammar. But see also Sampson (1999) and Tomasello (2005), among others.

4 Often UG is confused in the popular media with “Deep Structure,” perhaps owing in part to the work on the “Universal Base Hypothesis” of the Generative Semanticists, the forefathers of functionalism. Though many linguists laugh at this confusion, it is somewhat understandable.

5 This is not entirely true, of course. One can say, while dying in a movie, for example (in the indicative mood), “Must save Susan” or “Feel no hunger,” etc.

6 Note that specific theories which assume UG make many predictions, though none that UG itself is causally implicated in so far as I can tell.

7 But, in fact, there is not a shred of evidence for this. In fact, there is no evidence at all for any language-specific cognitive deficit (not even the Whorfian-labeled “Specific Language Impairment,” Everett, in progress).

8 Moreover, given what we know about “dual inheritance theory” and other examples of quick genetic changes due to cultural pressures, e.g., lactose tolerance in populations where milk is a part of the diet beyond infancy – a mutation that spread within the past 6,000 years, well within the time frame of, say, pro-drop (which goes back to Indo-European), there is nothing implausible about quick changes.

9 Much of the following is taken from Everett (accepted).

10 Sonority is a formal property of sounds in which it is easier to produce “spontaneous voicing (vibration of the vocal folds while producing the sound),” though the lay person can refer to sonority as relative loudness with little loss of accuracy.

11 For independent reasons – but reasons that once again show the inadequacy of the SSG, onsets are preferred to codas, thus favoring the syllabification of *o.pa* over *op.a*. The reason that a simple preference such as “prefer onsets” is a problem for the SSG is that the preference clearly shows that SSG is unable to provide an adequate theory of syllabification (at least on its own).

12 Formants are caused by resonance in the vocal tract. They are concentrations of acoustic energy around a particular frequency in the speech stream. Different formant frequencies and amplitudes result from changing shapes of the tract. For any given segment there will be several formants, each spaced at 1000 Hz intervals. By resonance in the vocal tract, I mean a place in the vocal apparatus where there is a space for vibration – the mouth, the lips, the throat, the nasal cavity, and so on.

14 Because Hauser et al. (2002) claim that recursion just is the sole item of the Narrow Faculty of Language (FLN) its absence in a language cannot be equated with the lack of clicks in English or the lack of more than three vowels in Hawaiian. Those who claim this kind of thing would be committing a category-mistake.
